# 6-Meth­oxy-*N*-methyl-3-nitro-4-nitro­methyl-4*H*-chromen-2-amine

**DOI:** 10.1107/S1600536811015595

**Published:** 2011-04-29

**Authors:** J. Muthukumaran, A. Parthiban, P. Manivel, H. Surya Prakash Rao, R. Krishna

**Affiliations:** a Centre for Bioinformatics, School of Life Sciences, Pondicherry University, Puducherry 605 014, India; b Department of Chemistry, Pondicherry University, Puducherry 605 014, India

## Abstract

In the title compound, C_12_H_13_N_3_O_6_, the dihydro­pyran ring adopts a near screw-boat conformation. The dihedral angle between the mean planes of the benzene and dihydro­pyran rings is 6.35 (5)°. An intra­molecular N—H⋯O hydrogen bond generates an *S*(6) motif, which stabilizes the mol­ecular conformation. In the crystal, weak inter­molecular C—H⋯O, N—H⋯O and C—H⋯π hydrogen bonds contribute to the stabilization of the packing.

## Related literature

For related structures, see: Gayathri *et al.* (2006 ▶); Bhaskaran *et al.* (2006 ▶). For the biological importance of 4*H-*chromene derivatives, see: Cai (2007 ▶, 2008 ▶); Cai *et al.* (2006 ▶); Gabor *et al.* (1988 ▶); Brooks (1998 ▶); Valenti *et al.* (1993 ▶); Hyana & Saimoto (1987 ▶); Tang *et al.* (2007 ▶); Wang *et al.* (2000 ▶). For ring puckering analysis, see: Cremer & Pople (1975 ▶). For C—H⋯π inter­actions, see: Desiraju & Steiner (1999 ▶).
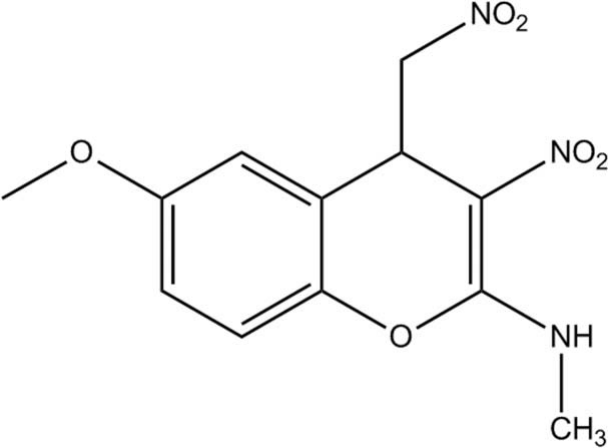


## Experimental

### 

#### Crystal data

C_12_H_13_N_3_O_6_
*M* *_r_* = 295.25
Monoclinic, 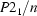

*a* = 6.8354 (2) Å
*b* = 9.4363 (2) Å
*c* = 19.9332 (4) Å
β = 90.777 (2)°
*V* = 1285.59 (5) Å^3^
*Z* = 4
Mo *K*α radiation
μ = 0.12 mm^−1^
*T* = 293 K
0.4 × 0.4 × 0.2 mm

#### Data collection

Oxford Diffraction Xcalibur Eos diffractometer
Absorption correction: multi-scan (*CrysAlis PRO*; Oxford Diffraction, 2009 ▶) *T*_min_ = 0.966, *T*_max_ = 1.000
13856 measured reflections
2256 independent reflections
1804 reflections with *I* > 2σ(*I*)
*R*_int_ = 0.040

#### Refinement

*R*[*F* ^2^ > 2σ(*F* ^2^)] = 0.041
*wR*(*F* ^2^) = 0.116
*S* = 1.01
2256 reflections
192 parameters
H-atom parameters constrained
Δρ_max_ = 0.28 e Å^−3^
Δρ_min_ = −0.36 e Å^−3^

### 

Data collection: *CrysAlis CCD* (Oxford Diffraction, 2009 ▶); cell refinement: *CrysAlis RED* (Oxford Diffraction, 2009 ▶); data reduction: *CrysAlis RED*; program(s) used to solve structure: *SHELXS97* (Sheldrick, 2008 ▶); program(s) used to refine structure: *SHELXL97* (Sheldrick, 2008 ▶); molecular graphics: *ORTEP-3 for Windows* (Farrugia, 1997 ▶) and *PLATON* (Spek, 2009 ▶); software used to prepare material for publication: *PLATON*.

## Supplementary Material

Crystal structure: contains datablocks I, global. DOI: 10.1107/S1600536811015595/hq2002sup1.cif

Structure factors: contains datablocks I. DOI: 10.1107/S1600536811015595/hq2002Isup2.hkl

Supplementary material file. DOI: 10.1107/S1600536811015595/hq2002Isup3.cml

Additional supplementary materials:  crystallographic information; 3D view; checkCIF report

## Figures and Tables

**Table 1:** Hydrogen-bond geometry (Å, °) *Cg* is the centroid of the C1–C6 benzene ring.

*D*—H⋯*A*	*D*—H	H⋯*A*	*D*⋯*A*	*D*—H⋯*A*
N1—H1⋯O2	0.86	2.01	2.6169 (17)	127
N1—H1⋯O3^i^	0.86	2.26	2.9808 (18)	142
C11—H11*A*⋯O2^ii^	0.97	2.49	3.4366 (19)	165
C10—H10*A*⋯*Cg*^iii^	0.96	2.61	3.548 (2)	164
C10—H10*C*⋯*Cg*^iv^	0.96	2.86	3.706 (2)	148

Symmetry codes: (i) 
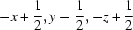
; (ii) 
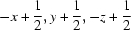
; (iii) 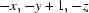; (iv) 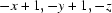.

**Fig. 1.**
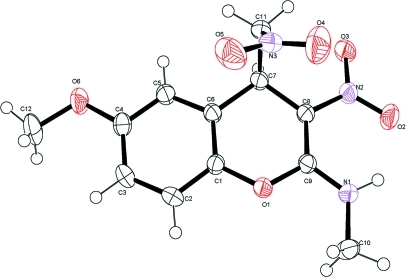
The molecular structure of (I), showing the atom-numbering scheme and displacement ellipsoids drawn at the 50% probability level.

**Fig. 2.**
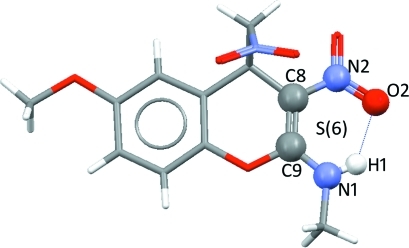
A view of the intramolecular S (6) motif formed by N—H···O interaction in Compound (I). The motif forming atoms are shown in ball and stick model and the hydrogen bond is shown as a blue dashed line.

**Fig. 3.**
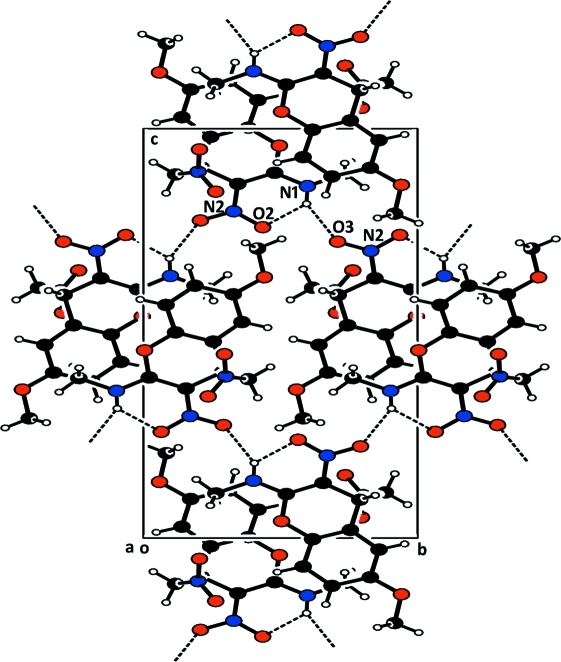
The crystal packing of Compound (I) viewed down the XO-axis, showing intermolecular hydrogen bonding interactions as dashed lines.

**Fig. 4.**
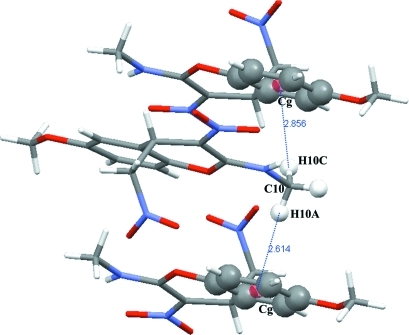
A view showing the weak C—H···pi intermolecular interactions in Compound (I). Cg is a centroid of the C1—C6 ring in the 4H-Chromene moiety.

**Fig. 5.**
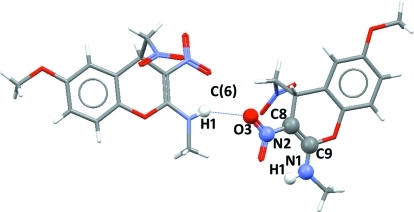
A view of the intermolecular C (6) motif formed by the N—H···O interaction in Compound (I). The motif forming atoms are shown in ball and stick model and the hydrogen bond is shown as a blue dashed line.

## supplementary crystallographic information

### Comment

4*H*-Chromene derivatives exhibit anti-viral, anti-fungal, anti-inflammatory, anti-diabetic, cardionthonic, anti-anaphylactic and anti-cancer activity (Cai, 2008; Cai, 2007; Cai *et al.*, 2006; Gabor *et al.*, 1988; Brooks, 1998; Valenti *et al.*, 1993; Hyana & Saimoto, 1987; Tang *et al.*, 2007). Functionally substituted 4*H*-Chromene derivatives are a new class of compound that binds to Bcl-2 protein and induces apoptosis or programmed cell death in cancer cells (Wang *et al.*, 2000). In order to examine the activity relationship between their molecular structure and biology, a single-crystal of the title compound was prepared for X-ray diffraction studies.

In the title compound (Fig. 1), the methoxy substituent at the C4 atom forms the torsion angle of -180 (14) ° [(-) anti-periplanar conformation] with the atom set O6/C4/C3/C2. From the puckering analysis (Cremer & Pople, 1975), the dihydropyran ring (O1/C1/C6/C7/C8/C9) is very similar to the screw-boat conformation (S form) with puckering parameters of Q = 0.1798 (15) Å, θ = 100.8 (5)° and Φ = 20.1 (5)°. Three intramolecular interactions N1—H1···O2 (symmetry code: *x*, *y*, *z*), N1—H1···N2 and C11—H11A···O3 are observed to contribute to the stability of the title compound, in which an N1—H1···O2 interaction generates a characteristic intramolecular *S* (6) motif with an N···O distance of 2.617 (17) Å (Fig. 2). The stabilization of crystal packing (Fig. 3) is influenced by intermolecular hydrogen bonding such as N1—H1···O3 (symmetry code: -*x* + 1/2, *y* - 1/2, -*z* + 1/2) and C11—H11A···O2 (symmetry code: -*x* + 1/2, *y* + 1/2, -*z* + 1/2). The C—H···pi interactions (Fig. 4) observed between C10—H10A···*Cg* (symmetry code:-*x*, 1 - *y*,-*z*, *Cg* is the centroid of the benzene ring C1—C6, C···*Cg* distance: 3.548 (2) Å, H-Perp: -2.56 Å) and C10—H10C···*Cg* (symmetry code: 1 - *x*,1 - *y*,-*z*, C···*Cg* distance: 3.706 (2) Å, H-Perp: 2.65 Å) also contribute to the crystal packing. The bond distances of the C—H···π interactions agree with those described by Desiraju & Steiner (1999). An intermolecular N1—H1···O3 interaction generates a *C* (6) motif with an N···O distance of 2.981 (18) Å (Fig. 5).

### Experimental

*(E)*-4-Methoxy-2-(2-nitrovinyl)phenol (200 mg, 1.024 mmol) was taken in a 25 ml round bottom flask in methanol (5 ml). To this solution, 1,8-diazabicyclo[5.4.0]undec-7-ene (DBU) (15 mg, 0.102 mmol) was added and stirred thoroughly for 10 minutes at room temperature. To this stirred solution, ((*E*) *N*-methyl-1-(methylthio)-2-nitroethenamine) (NMSM) was added and stirred for 8 h for completion (TLC, hexane:ethyl acetate, 3:2, *R*_f_ of I = 1/2). The reaction mixture was then kept in a refrigerator for 3 h to afford racemic mixture of the product (I)as a white precipitate, which was filtered. Good crystals were obtained by recrystallization with a solution of dichloromethane:hexane (9:3 *v*/*v*).

### Refinement

All hydrogen atoms were placed in calculated positions, with N—H = 0.86 and C—H = 0.97 and included in the final cycles of refinement using a riding model with *U*_iso_(H) = 1.2 *U*_eq_(C).

TITL

*CELL* 0.71073 6.8354 9.4363 19.9332 90.000 90.777 90.000

ZERR 4.00 0.0002 0.0002 0.0004 0.000 0.002 0.000

LATT 1

SYMM 1/2 - *X*, 1/2 + Y, 1/2 - *Z*

SFAC C H N O

UNIT 48 52 12 24

MERG 2

*OMIT* -2 50

ACTA

CONF

FMAP 2

PLAN 20

BOND $H

EQIV $1 1/2-*X*, -1/2+Y, 1/2-*Z*

EQIV $2 1/2-*X*, 1/2+Y, 1/2-*Z*

HTAB N1 O3_$1

HTAB C11 O2_$2

L.S. 4

WGHT 0.087000

FVAR 5.43518

### Crystal data

**Table:**

C_12_H_13_N_3_O_6_	*F*(000) = 616
*M**_r_* = 295.25	*D*_x_ = 1.525 Mg m^−^^3^
Monoclinic, *P*2_1_/*n*	Mo *K*α radiation, λ = 0.71073 Å
Hall symbol: -P 2yn	Cell parameters from 7594 reflections
*a* = 6.8354 (2) Å	θ = 3.0–29.3°
*b* = 9.4363 (2) Å	µ = 0.12 mm^−^^1^
*c* = 19.9332 (4) Å	*T* = 293 K
β = 90.777 (2)°	Block, colorless
*V* = 1285.59 (5) Å^3^	0.4 × 0.4 × 0.2 mm
*Z* = 4	

### Data collection

**Table:**

Oxford Diffraction Xcalibur Eos diffractometer	2256 independent reflections
Radiation source: fine-focus sealed tube	1804 reflections with *I* > 2σ(*I*)
graphite	*R*_int_ = 0.040
Detector resolution: 15.9821 pixels mm^-1^	θ_max_ = 25.0°, θ_min_ = 3.0°
ω scans	*h* = −8→8
Absorption correction: multi-scan (*CrysAlis PRO*; Oxford Diffraction, 2009)	*k* = −11→11
*T*_min_ = 0.966, *T*_max_ = 1.000	*l* = −23→23
13856 measured reflections	

### Refinement

**Table:**

Refinement on *F*^2^	Primary atom site location: structure-invariant direct methods
Least-squares matrix: full	Secondary atom site location: difference Fourier map
*R*[*F*^2^ > 2σ(*F*^2^)] = 0.041	Hydrogen site location: inferred from neighbouring sites
*wR*(*F*^2^) = 0.116	H-atom parameters constrained
*S* = 1.01	*w* = 1/[σ^2^(*F*_o_^2^) + (0.087*P*)^2^] where *P* = (*F*_o_^2^ + 2*F*_c_^2^)/3
2256 reflections	(Δ/σ)_max_ = 0.015
192 parameters	Δρ_max_ = 0.28 e Å^−^^3^
0 restraints	Δρ_min_ = −0.36 e Å^−^^3^

### Special details

**Table:**

Geometry. All e.s.d.'s (except the e.s.d. in the dihedral angle between two l.s. planes) are estimated using the full covariance matrix. The cell e.s.d.'s are taken into account individually in the estimation of e.s.d.'s in distances, angles and torsion angles; correlations between e.s.d.'s in cell parameters are only used when they are defined by crystal symmetry. An approximate (isotropic) treatment of cell e.s.d.'s is used for estimating e.s.d.'s involving l.s. planes.
Refinement. Refinement of *F*^2^ against ALL reflections. The weighted *R*-factor *wR* and goodness of fit *S* are based on *F*^2^, conventional *R*-factors *R* are based on *F*, with *F* set to zero for negative *F*^2^. The threshold expression of *F*^2^ > σ(*F*^2^) is used only for calculating *R*-factors(gt) *etc*. and is not relevant to the choice of reflections for refinement. *R*-factors based on *F*^2^ are statistically about twice as large as those based on *F*, and *R*- factors based on ALL data will be even larger.

### Fractional atomic coordinates and isotropic or equivalent isotropic displacement parameters (Å^2^)

**Table:**

	*x*	*y*	*z*	*U*_iso_*/*U*_eq_	
C1	0.2620 (2)	0.61723 (15)	−0.00254 (7)	0.0299 (4)	
C2	0.2708 (2)	0.58244 (17)	−0.06949 (8)	0.0337 (4)	
H2	0.2594	0.4884	−0.0830	0.040*	
C3	0.2967 (2)	0.68797 (18)	−0.11657 (8)	0.0356 (4)	
H3	0.3019	0.6656	−0.1620	0.043*	
C4	0.3149 (2)	0.82767 (17)	−0.09555 (8)	0.0335 (4)	
C5	0.3093 (2)	0.85951 (17)	−0.02791 (7)	0.0329 (4)	
H5	0.3239	0.9532	−0.0142	0.039*	
C6	0.2826 (2)	0.75487 (16)	0.01994 (7)	0.0297 (4)	
C7	0.2766 (2)	0.78954 (16)	0.09386 (7)	0.0323 (4)	
H7	0.3939	0.8453	0.1045	0.039*	
C8	0.2891 (2)	0.65553 (16)	0.13472 (7)	0.0310 (4)	
C9	0.2506 (2)	0.52117 (16)	0.10765 (7)	0.0289 (4)	
C10	0.2007 (2)	0.26444 (16)	0.10926 (9)	0.0379 (4)	
H10A	0.0798	0.2655	0.0842	0.057*	
H10B	0.1950	0.1928	0.1434	0.057*	
H10C	0.3067	0.2442	0.0796	0.057*	
C11	0.0974 (3)	0.88254 (16)	0.11164 (8)	0.0379 (4)	
H11A	0.1119	0.9171	0.1573	0.046*	
H11B	0.0916	0.9638	0.0819	0.046*	
C12	0.3430 (3)	0.9162 (2)	−0.20753 (8)	0.0543 (5)	
H12A	0.4498	0.8543	−0.2180	0.081*	
H12B	0.3585	1.0045	−0.2308	0.081*	
H12C	0.2219	0.8728	−0.2213	0.081*	
N1	0.2318 (2)	0.40166 (13)	0.14032 (6)	0.0336 (3)	
H1	0.2383	0.4048	0.1834	0.040*	
N2	0.3329 (2)	0.67051 (14)	0.20171 (6)	0.0356 (3)	
N3	−0.0864 (2)	0.80035 (15)	0.10518 (7)	0.0411 (4)	
O1	0.22704 (16)	0.50418 (11)	0.04132 (5)	0.0358 (3)	
O2	0.3398 (2)	0.56472 (13)	0.24035 (5)	0.0496 (4)	
O3	0.3669 (2)	0.79190 (12)	0.22406 (6)	0.0484 (4)	
O4	−0.1438 (2)	0.73633 (16)	0.15375 (8)	0.0703 (5)	
O5	−0.1686 (2)	0.79552 (16)	0.05057 (7)	0.0641 (4)	
O6	0.34105 (19)	0.94142 (13)	−0.13716 (6)	0.0486 (4)	

### Atomic displacement parameters (Å^2^)

**Table:**

	*U*^11^	*U*^22^	*U*^33^	*U*^12^	*U*^13^	*U*^23^
C1	0.0314 (8)	0.0310 (8)	0.0272 (8)	−0.0014 (6)	0.0001 (6)	0.0012 (7)
C2	0.0359 (9)	0.0344 (9)	0.0307 (9)	−0.0006 (7)	0.0012 (7)	−0.0079 (7)
C3	0.0352 (9)	0.0487 (10)	0.0230 (8)	−0.0021 (7)	0.0032 (7)	−0.0045 (7)
C4	0.0309 (8)	0.0418 (9)	0.0280 (8)	−0.0032 (7)	0.0031 (6)	0.0049 (7)
C5	0.0366 (9)	0.0321 (8)	0.0299 (8)	−0.0033 (7)	0.0004 (7)	0.0001 (7)
C6	0.0321 (8)	0.0318 (8)	0.0253 (8)	−0.0015 (6)	0.0000 (6)	−0.0011 (6)
C7	0.0439 (9)	0.0280 (8)	0.0250 (8)	−0.0055 (7)	−0.0029 (7)	−0.0021 (6)
C8	0.0381 (9)	0.0309 (8)	0.0238 (8)	−0.0002 (7)	−0.0020 (6)	−0.0013 (6)
C9	0.0297 (8)	0.0318 (8)	0.0253 (8)	0.0030 (6)	−0.0003 (6)	−0.0002 (6)
C10	0.0427 (10)	0.0285 (9)	0.0424 (10)	−0.0002 (7)	−0.0050 (8)	−0.0008 (7)
C11	0.0610 (11)	0.0249 (8)	0.0278 (9)	0.0013 (7)	−0.0020 (7)	−0.0042 (7)
C12	0.0661 (13)	0.0703 (14)	0.0267 (9)	−0.0128 (10)	0.0045 (8)	0.0082 (9)
N1	0.0443 (8)	0.0296 (7)	0.0270 (7)	0.0007 (6)	−0.0005 (6)	−0.0001 (6)
N2	0.0455 (8)	0.0358 (8)	0.0254 (7)	−0.0029 (6)	−0.0042 (6)	−0.0010 (6)
N3	0.0534 (9)	0.0347 (8)	0.0353 (9)	0.0109 (6)	0.0022 (7)	−0.0042 (7)
O1	0.0538 (7)	0.0289 (6)	0.0244 (6)	−0.0057 (5)	−0.0031 (5)	−0.0018 (4)
O2	0.0772 (9)	0.0417 (7)	0.0297 (7)	−0.0045 (6)	−0.0105 (6)	0.0074 (5)
O3	0.0775 (9)	0.0378 (7)	0.0297 (7)	−0.0097 (6)	−0.0054 (6)	−0.0087 (5)
O4	0.0762 (10)	0.0754 (10)	0.0597 (9)	−0.0071 (8)	0.0115 (8)	0.0222 (8)
O5	0.0679 (10)	0.0781 (10)	0.0460 (8)	0.0039 (7)	−0.0138 (7)	−0.0154 (7)
O6	0.0696 (9)	0.0479 (7)	0.0285 (6)	−0.0079 (6)	0.0065 (6)	0.0080 (5)

### Geometric parameters (Å, °)

**Table:**

C1—C2	1.376 (2)	C9—O1	1.3394 (17)
C1—C6	1.381 (2)	C10—N1	1.4496 (19)
C1—O1	1.4019 (17)	C10—H10A	0.9600
C2—C3	1.381 (2)	C10—H10B	0.9600
C2—H2	0.9300	C10—H10C	0.9600
C3—C4	1.388 (2)	C11—N3	1.480 (2)
C3—H3	0.9300	C11—H11A	0.9700
C4—O6	1.3696 (19)	C11—H11B	0.9700
C4—C5	1.382 (2)	C12—O6	1.423 (2)
C5—C6	1.387 (2)	C12—H12A	0.9600
C5—H5	0.9300	C12—H12B	0.9600
C6—C7	1.511 (2)	C12—H12C	0.9600
C7—C8	1.506 (2)	N1—H1	0.8600
C7—C11	1.551 (2)	N2—O3	1.2497 (17)
C7—H7	0.9800	N2—O2	1.2613 (16)
C8—N2	1.3719 (19)	N3—O4	1.2111 (18)
C8—C9	1.401 (2)	N3—O5	1.2191 (19)
C9—N1	1.3095 (19)		
			
C2—C1—C6	122.24 (14)	N1—C10—H10A	109.5
C2—C1—O1	115.67 (13)	N1—C10—H10B	109.5
C6—C1—O1	122.07 (13)	H10A—C10—H10B	109.5
C1—C2—C3	119.63 (14)	N1—C10—H10C	109.5
C1—C2—H2	120.2	H10A—C10—H10C	109.5
C3—C2—H2	120.2	H10B—C10—H10C	109.5
C2—C3—C4	119.43 (14)	N3—C11—C7	110.81 (12)
C2—C3—H3	120.3	N3—C11—H11A	109.5
C4—C3—H3	120.3	C7—C11—H11A	109.5
O6—C4—C5	115.20 (14)	N3—C11—H11B	109.5
O6—C4—C3	124.98 (14)	C7—C11—H11B	109.5
C5—C4—C3	119.81 (14)	H11A—C11—H11B	108.1
C6—C5—C4	121.45 (15)	O6—C12—H12A	109.5
C6—C5—H5	119.3	O6—C12—H12B	109.5
C4—C5—H5	119.3	H12A—C12—H12B	109.5
C1—C6—C5	117.41 (14)	O6—C12—H12C	109.5
C1—C6—C7	121.09 (13)	H12A—C12—H12C	109.5
C5—C6—C7	121.49 (13)	H12B—C12—H12C	109.5
C8—C7—C6	110.10 (12)	C9—N1—C10	124.87 (13)
C8—C7—C11	112.99 (13)	C9—N1—H1	117.6
C6—C7—C11	112.17 (12)	C10—N1—H1	117.6
C8—C7—H7	107.1	O3—N2—O2	120.17 (13)
C6—C7—H7	107.1	O3—N2—C8	118.59 (13)
C11—C7—H7	107.1	O2—N2—C8	121.24 (13)
N2—C8—C9	120.36 (13)	O4—N3—O5	123.00 (17)
N2—C8—C7	116.74 (13)	O4—N3—C11	118.40 (15)
C9—C8—C7	122.86 (13)	O5—N3—C11	118.53 (15)
N1—C9—O1	112.12 (13)	C9—O1—C1	120.35 (12)
N1—C9—C8	127.37 (14)	C4—O6—C12	117.96 (14)
O1—C9—C8	120.52 (13)		
			
C6—C1—C2—C3	−1.5 (2)	N2—C8—C9—N1	7.3 (2)
O1—C1—C2—C3	177.37 (13)	C7—C8—C9—N1	−170.06 (15)
C1—C2—C3—C4	0.5 (2)	N2—C8—C9—O1	−173.30 (13)
C2—C3—C4—O6	−179.99 (14)	C7—C8—C9—O1	9.4 (2)
C2—C3—C4—C5	0.8 (2)	C8—C7—C11—N3	−55.31 (17)
O6—C4—C5—C6	179.61 (14)	C6—C7—C11—N3	69.86 (16)
C3—C4—C5—C6	−1.1 (2)	O1—C9—N1—C10	3.5 (2)
C2—C1—C6—C5	1.2 (2)	C8—C9—N1—C10	−177.05 (15)
O1—C1—C6—C5	−177.60 (13)	C9—C8—N2—O3	179.81 (14)
C2—C1—C6—C7	−178.67 (14)	C7—C8—N2—O3	−2.7 (2)
O1—C1—C6—C7	2.5 (2)	C9—C8—N2—O2	0.0 (2)
C4—C5—C6—C1	0.1 (2)	C7—C8—N2—O2	177.50 (14)
C4—C5—C6—C7	179.97 (14)	C7—C11—N3—O4	89.78 (17)
C1—C6—C7—C8	11.8 (2)	C7—C11—N3—O5	−87.28 (17)
C5—C6—C7—C8	−168.03 (14)	N1—C9—O1—C1	−173.34 (12)
C1—C6—C7—C11	−114.91 (16)	C8—C9—O1—C1	7.2 (2)
C5—C6—C7—C11	65.23 (18)	C2—C1—O1—C9	167.88 (13)
C6—C7—C8—N2	164.64 (13)	C6—C1—O1—C9	−13.3 (2)
C11—C7—C8—N2	−69.08 (17)	C5—C4—O6—C12	−178.38 (14)
C6—C7—C8—C9	−17.9 (2)	C3—C4—O6—C12	2.3 (2)
C11—C7—C8—C9	108.34 (16)		

### Hydrogen-bond geometry (Å, °)

**Table:**

*Cg* is the centroid of the C1–C6 benzene ring.

**Table:**

*D*—H···*A*	*D*—H	H···*A*	*D*···*A*	*D*—H···*A*
N1—H1···O2	0.86	2.01	2.6169 (17)	127
N1—H1···O3^i^	0.86	2.26	2.9808 (18)	142
C11—H11A···O2^ii^	0.97	2.49	3.4366 (19)	165
C10—H10A···Cg^iii^	0.96	2.61	3.548 (2)	164
C10—H10C···Cg^iv^	0.96	2.86	3.706 (2)	148

Symmetry codes: (i) −*x*+1/2, *y*−1/2, −*z*+1/2; (ii) −*x*+1/2, *y*+1/2, −*z*+1/2; (iii) −*x*, −*y*+1, −*z*; (iv) −*x*+1, −*y*+1, −*z*.
